# Motivational regulations for exercise in Brazilian outpatients with bipolar disorder: An analysis based on the self-regulation theory

**DOI:** 10.1371/journal.pone.0354264

**Published:** 2026-07-28

**Authors:** Fernanda Castro Monteiro, Carlos Linhares Veloso Filho, Thaís de Almeida Britto, Clara Moreira Zettel, Laíne Keisy Siqueira da Silva, Felipe Barreto Schuch, Elie Cheniaux, Thiago Sousa Matias, Lara Carneiro, Andrea Deslandes

**Affiliations:** 1 Psychiatry Institute, Federal University of Rio de Janeiro, Rio de Janeiro, Brazil; 2 Institute of Health Sciences, Universidad de O’Higgins, Rancagua, Chile; 3 Department of Sports Methods and Techniques, Federal University of Santa Maria, Santa Maria, Brazil; 4 Faculty of Health Sciences, Universidad Autónoma de Chile, Providência, Chile; 5 Institute for Physical Activity and Nutrition, Deakin University, Geelong, Australia; 6 Institute for Mental and Physical Health and Clinical Translation (IMPACT), Deakin University, Geelong, Australia; 7 Faculty of Medical Sciences, State University of Rio de Janeiro, Rio de Janeiro, Brazil; 8 Federal University of Santa Catarina, Florianopolis, Brazil; 9 Physical Education Department, College of Education, United Arab Emirates University, Al Ain, Abu Dhabi, United Arab Emirates; ESECS-Polytechnique of Leiria / Research Center in Sports Sciences, Health Sciences and Human Development, CIDESD, PORTUGAL

## Abstract

Despite the well-known physical activity (PA) benefits for physical and mental health, bipolar patients seem to be insufficiently active and often encounter challenges in participating in and adhering to programs involving PA. The present study aimed to assess the motivational regulation for exercise in bipolar disorder patients. This cross-sectional study utilizes objective (accelerometers) measures to assess PA. The Young Mania Rating Scale (YMRS) and the Hamilton Depression Rating Scale (HAM-D) were used to assess mania and depressive symptoms. The Behavioral Regulation in Exercise Questionnaire – 3 (BREQ-3) was used to assess the motivators for exercise. The sample was composed of 43 patients with bipolar disorder (81.5% female, mean age = 47 years; SD = 10.4). Sufficiently actives patients according to accelerometer moderate-vigorous physical activity (MVPA) were significantly more intrinsically regulated than those insufficiently actives (p = 0.002). The linear regression model showed that intrinsic regulation predicted 20% of the variability for accelerometer MVPA (p = 0.004). Notable contrasts were observed in integrated regulation. When asked whether they consider exercise as a part of their identity, 27.6% of the insufficiently active patients selected “Very true for me.” Meanwhile this view was endorsed by 57.1% of sufficiently active. Similarly, 50% of the active group reported that exercise is a fundamental part of who they are compared with 31% of the insufficiently active group. Our findings suggest that more autonomous forms of motivation, particularly integrated and intrinsic regulation, were associated with higher levels of PA among individuals with BD, whereas less active participants tended to present higher depressive symptomatology and lower motivational quality.

## 1. Introduction

The life expectancy of individuals with bipolar disorder (BD) is approximately 12–14 years shorter than that of general population due to multifactorial causes [1,2]. Factors such as cardio-metabolic disease, poor lifestyle habits, and suicide risk play an important role in the increased premature mortality among this population [3]. Despite the well-known physical activity (PA) benefits for physical and mental health, bipolar patients seem to be insufficiently active and excessively sedentary [4,5].

Moreover, they often encounter challenges in participating and adhering to programs involving PA and exercise [6]. These difficulties stem from various factors such as perceived stress, somatic comorbidities, depressed mood, and lack of self-confidence and lack of social support [6]. However, understanding the motivational process involved in PA behavior more broadly in bipolar individuals seems to be still unclear.

Deci and Ryan’s Self-Determination Theory (SDT) posits that behavior is regulated by different forms of motivation which can be characterized by the degree of self-determination [7]. Rooted in a humanistic perspective and focused on the fulfillment of basic psychological needs, SDT suggests that people are motivated by three basic psychological needs: autonomy (the feeling of control over your own actions); competence (the feeling of being capable and effective in what you do) and relatedness (the feeling of connection and belonging with others). While practicing PA, when these needs are met, motivation tends to be more intrinsic (pleasure and satisfaction in carrying out the activity). Someone who feels competent and autonomous when exercise is more likely to maintain this activity in the long term. In contrast, amotivation refers to the absence of any intention to act or engage in a specific behavior. Extrinsic motivation refers to engaging in an activity for reasons external to the self, such as rewards, pressures or obligations [7].

Previous studies involving behavior change literature have highlighted the important role of motivation in engaging and maintaining an active lifestyle in individuals facing mental illness [8,9]. In a recent study with mental illness patients – the majority from higher-income countries – autonomous motivation was associated with 31% higher MVPA, while amotivation were associated with 18% lower MVPA [10]. An Australian study of two exercise interventions (GYM – participated in an exercise instruction program or MOT – received behavioral counseling) for adults with mental illness, measured self-determined motivation, physical health indicators and mental health at baseline and post-intervention [11]. Participants in the GYM group showed significant improvements in self-determined motivation and experienced reductions in psychological distress, indicating a relationship between enhanced autonomous motivation and mental health benefits.

While the benefits of PA for mental health are well-documented [12], the specific motivational mechanisms driving exercise behavior in individuals with BD remain poorly understood. Most existing literature on motivational regulation focuses on general mental illness or major depressive disorder [13], often overlooking the unique clinical challenges of BD, such as the fluctuating nature of mood episodes (mania, hypomania, and depression) and their distinct impacts on self-regulatory processes. There is a critical gap in understanding how the continuum of self-determination – ranging from amotivation to intrinsic motivation – operates within the complex symptomatic profile of BD. Specifically, it remains unknown how autonomous forms of motivation (intrinsic and integrated regulation) relate to objectively measured PA levels in this population, particularly in low- and middle-income contexts like Brazil, where environmental and social barriers to exercise are more pronounced. Considering this gap in literature, our study seeks to utilize the SDT to understand the motivational process involved in exercise behavior among BD outpatients. Based on SDT and the existing literature, we propose the following hypotheses: Patients with BD who achieve the recommended levels of PA will exhibit significantly higher levels of autonomous motivation (intrinsic and integrated regulation) compared to those who do not achieve these levels; Autonomous motivation (intrinsic and integrated regulation) will be positively associated with levels of MVPA measured by accelerometry in patients with BD.

In light of this, the present study aims to investigate motivators to exercise in this population utilizing the Behavioral Regulation in Exercise Questionnaire-3 (BREQ-3) [14], commonly used to measure the different motivational subtypes for exercising. Second, we aimed to investigate if different motivation types are associated with current PA levels of people with BD. Third, we aim to compare the motivational regulations among individuals who reached and did not reach the recommendations of the PA guidelines according to PA measures [14].

## 2. Methods

This is a cross-sectional study. The sample was composed of individuals of both sexes, aged between 28 and 64 years, diagnosed with a mental illness and undergoing outpatient treatment at the Institute of Psychiatry of the Federal University of Rio de Janeiro (IPUB/UFRJ). Patients were invited to participated randomly by our staff at the Bipolar Disorder Outpatient Clinic at the Institute of Psychiatry IPUB/ UFRJ. Sampling was conducted on a convenience basis, with patients invited sequentially by the researchers involved in the study and by the staff of the Bipolar Disorder Outpatient Clinic at the Institute of Psychiatry of the Federal University of Rio de Janeiro (IPUB/UFRJ) during their routine appointments. Patients had a diagnosis of BD type I or II according to DSM-IV criteria. The participation rate was approximately 40%. That is, for every 10 participants invited to take part in the study, 4 agreed to participate. The sample size was calculated based on the study titled: “Are adults with bipolar disorder active? Objectively measured physical activity and sedentary behavior using accelerometry” [15]. We performed a statistical power calculation for multiple linear regression (G*Power 3.1.9.7), using an effect size (f²) of 0.50, a significance level (α) of 0.05, and a statistical power of 0.95 for four predictors. This calculation indicated a required sample size of 43 participants, which corresponds exactly to the number of participants included in the study.

The participants were not enrolled in any structured PA program at the Bipolar Disorder Outpatient Clinic. Additionally, variables such as prior PA experience or engagement in routine PA were not controlled. The inclusion criteria were as follows: individuals of either sex, aged 18–65, diagnosed with a mental illness and receiving outpatient treatment at the Institute of Psychiatry of the Federal University of Rio de Janeiro (IPUB/UFRJ), who had received medical clearance to engage PA. Exclusion criteria included patients with mobility impairments, severe physical comorbidities that could prevent them from safely engaging in PA, people who do not have a medical recommendation to engage in PA, or incomplete accelerometer data. In this study, one participant had to be excluded on the basis of incomplete accelerometer data, and two participants were excluded due to the loss of the accelerometer. Data collections were conducted between March 2022 and December 2023. Precisely, it started in 25/03/2022 and finished in 20/12/2023. The research protocol was approved by the Research Ethics Committee of the Institute of Psychiatry at the Federal University of Rio de Janeiro (IPUB/UFRJ), under registration CAAE: 48245021.7.0000.5263.

All participants provided written informed consent prior to data collection. To ensure data confidentiality and anonymization, all personal information (name, address, phone number) was collected separately from the research data and stored in a secure database accessible only to authorized researchers. The research data was coded with unique identifiers, dissociating them from any personal information, thereby ensuring anonymity in analyses and publications.

After signing the Informed Consent Form, participants completed the following evaluations: anamnesis containing sociodemographic questions; application of the *Mini International Neuropsychiatric Interview (M.I.N.I. Plus)*; *Hamilton Depression Rating Scale (HAM-D)*; *Young Mania Rating Scale (YMRS)*; application of the *Behavioral Regulation in Exercise Questionnaire – 3 (BREQ-3)*; placing the accelerometer *(ActiGraph wGT3X-BT)* on participants right hip during waking hours for 7 consecutive days.

All the questionnaires were administered by trained interviewers, with an average completion time of 80 minutes, and were completed after the signing of the written consent form and before the accelerometer was attached. Internal consistency analyses were performed for symptom severity scales and BREQ-3 scale using Cronbach’s alpha coefficients. Cronbach’s alpha coefficients in the present sample were 0.79 for the HAM-D, 0.74 for the YMRS, and 0.72 for the BREQ-3 scale.

### 2.1. Instruments

#### 2.1.1. Anamnesis

This process gathers personal and sociodemographic data, such as name, address, telephone number, date of birth, sex, marital status, education, income, health history, medication usage, and PA practice. At this stage, information was collected on medication use and the type of BD (Type I or Type II) according to the patients’ medical records and the responses obtained during the interview.

#### 2.1.2. Mini International Neuropsychiatric Interview (M.I.N.I. Plus)

The MINI is a brief standardized diagnostic interview, compatible with DSM-III-R/IV and ICD-10 criteria, which is intended for use in clinical practice and research in primary care and psychiatry. Can be used by clinicians after initial training (1–3 hours). The more detailed Plus version of MINI generates positive diagnoses of the main DSM-IV psychotic and mood disorders [16].

#### 2.1.3. Hamilton Depression Rating Scale (HAM-D)

The HAM-D is a scale for assessing depressive symptoms. It features 17 items, which are evaluated within a determined period of days according to the intensity and frequency of the patient’s symptoms using a scale that varies from “absent” to “quite severe”. The scale ranges from 0 to 56 points, with scores above 23 indicating severely depressed patients; scores between 19 and 22 points, moderately depressed patients; between 14 and 18 points, mildly depressed patients, and scores lower than 7, euthymic patients [17].

#### 2.1.4. Young Mania Rating Scale (YMRS)

The Young Mania Rating Scale consists of 11 items and is one of the most commonly used instruments for evaluating manic symptoms. It includes both the patient’s subjective account of their clinical state and the clinician’s objective assessment of behavior over the past 48 hours. Each item is rated across levels of severity: seven items (elevated mood, increased motor activity/energy, sexual interest, sleep, language/thought disorder, appearance, and insight) are scored from 0 to 4, while the remaining four (irritability, speech, thought content, and disruptive/aggressive behavior) are scored from 0 to 8 [18].

#### 2.1.5. Behavioral Regulation Exercise Questionnaire – 3 (BREQ-3) (Brazilian version)

The BREQ-3 is a questionnaire based on the constructs of SDT. It postulates that the motivation to assume specific conditions may vary along a continuum according to the perceived degree of self-determination, which is based on the three basic psychological needs: autonomy, competence and relationships/relatedness [14]. At one end of the self-determination continuum is the construct called amotivation, which involves the absence of any type of motivation (intrinsic or extrinsic) and signifies the lowest degree of self-determination. At the other end of the continuum is intrinsic motivation, the most self-determined form of motivation, which involves practicing PA voluntarily for pleasure and satisfaction. Between the two poles (amotivation and intrinsic motivation) is extrinsic motivation, which is driven by external demands or rewards. Introjected regulation is driven by internal pressures like guilt or shame, whereas identified regulation occurs when the individual engages in the activity because it is personally important. Integrated regulation aligns the activity with personal identity and values, even as it still produces extrinsic outcomes, becoming part of an individual’s lifestyle. The Brazilian version of the BREQ-3 consists of 23 items, with respondents indicating their degree of agreement on a five-point Likert-type measurement scale ranging from 0 to 4 (0 = Not true for me; 4 = Very true for me). The questionnaire presents six motivation scales: (a) amotivation—AMOT; (b) external regulation—REEX; (c) introjected regulation—REIJ; (d) identified regulation—REID; (e) integrated regulation—REIG; and (f) intrinsic motivation—MOTI [14]. In the present study, in addition to presenting the scale scores in the usual manner, we chose to analyze the scale in a slightly different way, presenting the percentages of agreement and disagreement for each group (insufficiently active vs. sufficiently active) on each item of the BREQ-3.

#### 2.1.6. Accelerometer (ActiGraph wGT3X-BT)

Participants wore a triaxial accelerometer (ActiGraph®, wGT3X-BT model, Pensacola, FL, USA) that captures measures include gross acceleration, activity counts, energy expenditure, MET rates, steps taken, PA intensity, sedentary time. In the present study, the participants were asked to wear the accelerometer on their right hip – attached with an elastic belt – during waking hours for seven consecutive days, and to follow their regular daily routine. They were asked to remove the accelerometer only to perform water activities (i.e., showering, swimming) and sleep. The ActiGraph wGT3X-BT was initialized using the software ActiLife v6.13.3 with participants personal information. After participants returned the accelerometers, the data recorded by the devices were downloaded and processed using ActiLife software. The sampling frequency was 60 Hertz, in accordance with previous evidence [19,20]. The software converts raw accelerometer signals into summary data, commonly referred to as “epoch” data, which are subsequently processed through specific algorithms to generate activity outputs. Raw acceleration data were grouped into time intervals called epochs, and gravitational values (G values) were transformed into activity counts, representing both the intensity and frequency of movement within each epoch. These activity counts constitute the primary unit used by ActiLife to estimate PA levels. The filtering procedures used to generate activity counts are proprietary to ActiGraph and include embedded filters designed to remove signals outside the range of typical human movement, such as environmental vibrations, small tremors, and rapid accelerations associated with motorized transportation. Following data processing in ActiLife, accelerometer wear time was validated using the Troiano algorithm [21]. This algorithm identifies patterns in the accelerometer data to distinguish periods of wear and non-wear time, excluding intervals classified as non-use from subsequent analyses. Non-wear time was defined as periods of consecutive zero counts lasting 60 minutes or longer. To be considered valid for inclusion in the analyses, participants were required to provide at least four valid monitoring days, including a minimum of three weekdays and one weekend day, with at least 8 hours of accelerometer wear time per day, in accordance with previous recommendations [22–24]. In the present study, the following cut-points were used to classify activity intensity levels: sedentary behavior (<100 counts/minute), light-intensity physical activity (100–1951 counts/minute), and moderate-to-vigorous physical activity (MVPA ≥1952 counts/minute) [25–27]. For the cut-off based evaluation, we used an epoch length of 60 s epochs [19,20,28].

### 2.2. Data analysis

To analyze the normality and homoscedasticity of the data, the Shapiro Wilk and Levene’s test were conducted, respectively. Descriptive analyses were described using means and standard deviations for continuous measures and frequencies for categorical variables. To measure the association between variables, we performed linear regression models. Linear regression was adjusted for age and sex. To verify the differences in motivation scores between insufficiently and sufficiently actives individuals according to accelerometer measures, t-tests were performed. Effect sizes were calculated and reported for all relevant analyses (Cohen’s d for t-tests and Cohen’s f² for regressions), along with the rationale for the choice of statistical tests. We used the Statistical Package for the Social Sciences (SPSS 26.0, Armonk, NY, USA) to perform the statistical analyses.

## 3. Results

The sample was composed of 43 patients with BD, majority female (81.5%), 42% with monthly income of up to 3 minimum wages (low income), mean age = 47 years (SD = 10.4), 32.5% practiced PA, mean body mass index (BMI) = 30.7 (SD = 5.4), indicating a high prevalence of obesity (full details in Table 1). A total of 39 patients were classified as type 1, and four as type II BD. Most participants were using combined pharmacological treatment with mood stabilizers and antipsychotics (51.2%), followed by mood stabilizer monotherapy (23.3%) and antipsychotic monotherapy (18.6%). Smaller proportions used antidepressants alone (2.3%) or combined antidepressant and antipsychotic therapy (4.7%).

**Table 1:** Demographic characteristics

	Total sample (n = 43)
**Sex – n (%)**	
Female	35 (81.5%)
Male	8 (18.5%)
**Age – mean (SD)**	47 (10.40)
**Body mass index (BMI) – mean (SD)**	30.8 (5.42)
**Income range – n (%)**	
1–3 minimum wages	27 (64.2%)
3–6 minimum wages	11 (26.2%)
6–9 minimum wages	2 (4.8%)
More than 9 minimum wages	2 (4.8%)
**Marital status – n (%)**	
Single	18 (42%)
Married	11 (25.5%)
Divorced	11 (25.5%)
Widowed	1 (2.5%)
Did not inform	2 (4.5%)
**Education – n (%)**	
Postgraduate	7 (16.3%)
Incomplete college	7 (16.3%)
Complete college	7 (16.3%)
Complete high school	11 (25.5%)
Incomplete high school	4 (9.3%)
Complete elementary school	1 (2.3%)
Incomplete elementary school	6 (14%)
**Color/race**	
Black/brown	23 (53.5%)
White	20 (46.5%)
**Mood states – n (%)**	
Euthymia	17 (39.5%)
Mania	11 (25.5%)
Depression	15 (35%)
**Young Mania Rating Scale score – mean (SD)**	
Euthymia	2.94 (4.40)
Mania	11.82 (2.08)
Depression	2.87 (2.66)
**Hamilton Rating Scale for Depression score – mean (SD)**	
Euthymia	3.94 (3.92)
Mania	6.91 (4.48)
Depression	13.60 (5.85)
**Medication type – N (%)**	
Antidepressants	1 (2.3)
Antidepressants and antipsychotics	2 (4.7)
Antipsychotics	8 (18.6)
Mood stabilizers	10 (23.3)
Mood stabilizers and antipsychotics	22 (51.2)
**Accelerometer measures – mean (SD)**	
MVPA – minutes/week	136.91 (107.30)
Sedentary time – minutes/day	377.17 (193.93)
**BREQ-3 scores – mean (SD)**	
Amotivation	0.46 (0.82)
External regulation	1.27 (0.79)
Introjected regulation	2.22 (1.34)
Identified regulation	3.02 (0.88)
Integrated regulation	2.58 (1.25)
Intrinsic regulation	3.13 (10.03)

According to accelerometer measures, 14 patients met the recommended guidelines of PA, performing at least 150 minutes/week of MVPA, while 29 patients did not meet the recommended guidelines. According to HAM-D and YMRS scores, a total of 17 patients were categorized as euthymic, 11 as manic, and 15 as depressive (see Table 1).

Regarding mood states and PA levels, among the sufficiently active patients, 6 were manic (43%), 5 were euthymic (35.5%), and 3 were depressive (21.5%) (see Table 2). Among those who did not meet the PA recommendations, 12 were euthymic (41.5%), 12 were depressive (41.5%), and 5 were manic (17%).

**Table 2:** Distribution of mood states by level of physical activity

Mood states	Insufficiently actives MVPA < 150 min/week Accelerometer (n = 29)	Sufficiently actives MVPA ≥ 150 min/week Accelerometer (n = 14)
Euthymia	12 (41.5%)	5 (35.5%)
Mania	5 (17%)	6 (43%)
Depression	12 (41.5%)	3 (21.5%)

As can be noted in Table 2, sufficiently actives were significantly more intrinsic regulated than those who were insufficiently actives (see Table 3).

**Table 3:** Differences in motivation between insufficiently and sufficiently actives patients according to accelerometer (objective measures)

	Insufficiently actives MVPA < 150 min/week Accelerometer (n = 29)	Sufficiently actives MVPA ≥ 150 min/week Accelerometer (n = 14)	*P-value*	*Cohen’s d*
Amotivation – means (SD)	0.48 (0.64)	0.46 (0.91)	0.646	−0.03
External regulation – means (SD)	1.40 (0.92)	1.57 (1.09)	0.179	0.17
Introjected regulation – means (SD)	2.17 (1.39)	2.33 (1.28)	0.842	0.12
Identified regulation – means (SD)	3.01 (0.76)	2.93 (1.10)	0.953	−0.09
Integrated regulation – means (SD)	2.39 (1.27)	2.95 (0.94)	0.466	0.48
Intrinsic regulation – means (SD)	2.95 (1.01)	3.66 (0.61)	0.002*	0.79

MVPA = moderate-vigorous physical activity * = t-test with significance level set at p < 0.05. ** = t-test with significance level set at p < 0.001.

Considering BREQ-3 and PA levels, the linear regression model showed that the intrinsic regulation was associated with 20% of the variability for accelerometer active time MVPA (p = 0.004). The other variables on the BREQ-3 scale were not associated with active time (see Table 4).

**Table 4:** Linear regression analyses adjusted by sex and age – active time and BREQ-3 scores

Dependent variable	Independent variables	R²	Cohen’s *f*²	β	*p – value*	CI 95%	
						Lower	Upper
Active time MVPA (min/week)	Amotivation	0.008	0.008	−0.209	0.943	−6.089	5.671
	External regulation	0.023	0.024	2.377	0.452	−3.951	8.705
	Introjected regulation	0.011	0.011	−0.633	0.728	−4.294	3.028
	Identified regulation	0.023	0.024	2.136	0.444	−3.446	7.719
	Integrated regulation	0.065	0.070	2.981	0.132	−0.936	6.898
	Intrinsic regulation	0.203*	0.255	7.506	0.004*	2.584	12.427

CI: confidence interval; β: partial regression coefficient; R2: coefficient of determination. Significance level set at p < 0.05*.

Regarding amotivation statements, both groups presented a general pattern of disagreement (see Table 5 and S1 Fig). External regulation presented similar average scores across both groups. For the statement “I take part in exercise because my friends/family say I should,” 51.7% of the insufficiently active individuals responded “Very true for me,” compared with 42.9% of the sufficiently active participants, highlighting the influence of social encouragement, particularly among those less active individuals (see Table 5 and S2 Fig). This analysis was considered exploratory.

**Table 5:** Prevalence agreement of the Behavioral Regulation in Exercise Questionnaire-3 (BREQ-3) items in patients with bipolar disorder (n = 43)

BREQ-3 scale items	Insufficiently actives MVPA < 150 min/week Accelerometer (n = 29)		Sufficiently actives MVPA ≥ 150 min/week Accelerometer (n = 14)	
	(%) “Not true for me”	(%) “Very true for me”	(%) “Not true for me”	(%) “Very true for me”
**Amotivation**				
I don’t see why I should have to exercise	89.5%	3.5%	71.5%	7%
I don’t see the point in exercising	79.3%	6.9%	93%	7%
I can’t see why I should bother exercising	73%	13.8%	71.4%	14.3%
I think exercising is a waste of time	89.7%	0	93%	0
**Amotivation total**				
**External regulation**				
I exercise because other people say I should	72.4%	13.8%	78.6%	0
I take part in exercise because my friends/family say I should	13.8%	51.7%	14.3%	42.9%
I exercise because others will not be pleased with me if I don’t	69%	13.8%	42.9%	28.6%
I feel under pressure from my friends/family to exercise	58.6%	17.2%	42.9%	21.4%
**External regulation total**				
**Introjected regulation**				
I feel guilty when I don’t exercise	27.6%	51.7%	14.3%	42.9%
I feel ashamed when I miss an exercise session	51.7%	27.6%	42.9%	28.6%
I feel like a failure when I haven’t exercised in a while	27.6%	44.8%	28.6%	50%
**Introjected regulation total**				
**Identified regulation**				
It’s important for me to exercise regularly	10.3%	69%	14.3%	71.4%
I value the benefits of exercise	3.4%	79.3%	7.1%	78.6%
I think it’s important to make an effort to exercise regularly	3.4%	72.5%	14.3%	57.1%
I feel anxious if I don’t exercise regularly	48.3%	31.%	35.7%	35.7%
**Identified regulation total**				
**Integrated regulation**				
I exercise because it is consistent with my life goals	27.7%	44.8%	14.3%	57.2%
I consider exercise part of my identity	31%	27.6%	7.1%	57.1%
I consider exercise a fundamental part of who I am	31%	31%	14.3%	50%
I consider exercise consistent with my values	3.4%	62.1%	7.1%	71.5%
**Integrated regulation total**				
**Intrinsic motivation**				
I exercise because it’s fun	7.1%	46.6%	0	71.4%
I enjoy my exercise sessions	24.1%	51.7%	0	78.3%
I find exercise a pleasurable activity	6.9%	58.7%	0	71.4%
I get pleasure and satisfaction from participating in exercise	3.4%	75.9%	0	85.8%
**Intrinsic motivation total**				

Responses related to introjected regulation showed a comparable distribution between the two groups (see Table 5 and S3 Fig). With regard to identified regulation, the statement “I think it’s important to make an effort to exercise regularly” received strong agreement from 72.4% of the insufficiently active group and 57.1% of the sufficiently active group (see Table 5 and S4 Fig), suggesting that even less active individuals acknowledge the value of regular exercise.

More striking contrasts were observed in integrated regulation. When asked whether they consider exercise as a part of their identity, only 27.6% of the insufficiently active patients selected “Very true for me.” Meanwhile this view was endorsed by 57.1% of sufficiently active

patients, which is more than double. Similarly, 50% of the active group reported that exercise is a fundamental part of who they are compared with 31% of the insufficiently active group (see Table 5 and S5 Fig).

The most distinct difference was observed in intrinsic motivation. None of the sufficiently active patients marked “Not true for me” in any of the intrinsic motivation items. In contrast, 24.1% of the insufficiently active patients did not enjoy their sessions. While 46.4% of the insufficiently active group reported exercising because it is fun, this response was given by 71.4% of the sufficiently active patients. Likewise, only 51.7% of the less active group reported enjoying their sessions compared with 78.6% of the active group. A total of 58.6% of the insufficiently active patients strongly agreed to the item “I find exercise a pleasurable activity,” while the number rose to 71.4% among the active group. Finally, when asked whether they gain pleasure and satisfaction from exercise, 85.7% of the active group answered “Very true for me” and only 75.9% of their less active counterparts (see Table 5 and S6 Fig).

## 4. Discussion

To the best of our knowledge, this is the first Brazilian study to explore motivational regulation for exercise among patients with BD. Specifically, we compared insufficiently actives patients with those sufficiently actives, according to the recommended PA guidelines. Our findings indicate significant differences in intrinsic regulation between these groups, particularly when considering accelerometer measurements, aligning with previous research [29]. Regarding the motivation scores of the BREQ-3 scale, our data revealed that intrinsic regulation was associated with active time. Similar findings have been reported in studies involving individuals with mental illness, demonstrating a positive association between PA levels and autonomous motivation [29,30].

Considering medication use in our sample, the predominance of combined treatment involving mood stabilizers and antipsychotics may be clinically relevant when interpreting motivational outcomes related to PA, since these medications are frequently associated with sedation, fatigue, metabolic adverse effects, and reduced behavioral activation in individuals with BD [31]. In the same way, recent evidence suggests that psychotropic medication profiles may influence energy regulation, daily activity patterns, and engagement in health behaviors, indicating a bidirectional relationship between PA, clinical symptoms, and pharmacological treatment [32]. Most participants in the present study were diagnosed with BD type I, while only a small proportion presented BD type II. Previous studies have shown important differences between BD subtypes regarding symptom presentation, depressive burden, illness course, and functional outcomes [33–35]. Individuals with BD-II tend to experience greater depressive predominance, less euthymic episodes and mood instability, whereas BD-I is more frequently associated with manic episodes [34,35]. However, due to the limited number of participants with BD-II in the present sample, subgroup analyses were not feasible, and the findings should therefore be interpreted predominantly in the context of BD-I.

Our findings also showed that depressive symptoms were more prevalent among insufficiently active participants, indicating that mood-related clinical characteristics could have influenced motivational outcomes in the present study. This result is consistent with previous evidence demonstrating that depressive symptoms in individuals with BD are associated with reduced energy, fatigue, lower behavioral activation, poorer functioning, and decreased engagement in PA behaviors [32,36]. Studies examining the bidirectional relationship between mood symptoms and PA suggest that increases in PA levels are associated with reductions in depressive symptoms, while greater depressive symptom severity may contribute to lower engagement in PA behaviors [32]. Therefore, the higher prevalence of depressive symptoms among insufficiently active participants should be carefully considered when interpreting the motivational profiles observed in the present study, as depressive symptomatology itself may partially explain lower motivation and reduced engagement in PA.

With regard to amotivation statements, both groups presented a general pattern of disagreement. In terms of external regulation, both groups responded similarly regarding the influence of family and friends on PA participation, reinforcing the importance of social support in engagement [6,37]. This finding helps to elucidate and characterize the concept of external regulation proposed by the SDT, which refers to behavior that is motivated by external contingencies, such as rewards, approval, or pressure from others [7]. From this perspective, individuals may not engage in PA out of personal choice or intrinsic interest but because they are compelled to meet expectations or maintain relationships.

Introjected regulation, characterized by internal pressures such as guilt or obligation, was similarly distributed between the two groups, suggesting that while participants did not express high concern over missing individual sessions, they did resonate with the feeling of failure when their routines were interrupted. This may reflect issues related to self-worth, perceived competence, or fear of judgment – factors that can be particularly salient in mental health conditions [38,39].

In terms of identified regulation, our data indicated that insufficiently active patients perceived greater effort to exercise as requiring than their sufficiently active counterparts. This finding aligns with prior evidence suggesting that BD patients face numerous barriers to PA, including psychological, social, and logistical obstacles [39]. Moreover, both groups recognized the importance, benefits, and value of exercise. For those who are sufficiently active because exercise is more closely linked to their identity than simply being perceived as beneficial. For those who are insufficiently active, the recognition of the importance and benefits of exercise seems to be based on common sense rather than personal experience, since they do not comply with PA recommendations. This trend is in line with the literature, since participants with mental illnesses generally consider PA and exercise to be positive, but face difficulties in maintaining sufficient levels of PA [40]. This divergence between the two groups suggests differing levels of internalization, a key process in SDT through which externally endorsed values are gradually integrated into the self [7].

Stronger contrasts emerged in integrated regulation, which reflects the highest level of extrinsic motivation internalization, when behaviors are fully aligned with an individual’s identity and values. More than twice as many sufficiently active patients reported perceiving exercise as part of their identity compared with their insufficiently active peers. The active group also perceived exercise as a fundamental part of themselves. These findings reinforce the notion that integrated regulation is essential for long-term behavior change and lifestyle maintenance, especially when PA is experienced as congruent with self-concept [7,41]. The literature on physical literacy and early exposure to PA also supports this pattern – individuals who develop PA habits during childhood are more likely to maintain them in adulthood [41], with positive implications for social, psychological, and physical well-being [30]. The notable differences in integrated regulation between active and inactive patients in our study warrant further exploration through the lens of PA Identity. Recent evidence suggests that integrated regulation – where exercise is synonymous with how an individual views themselves – acts as a critical axis for behavioral stability [42]. In the context of BD, this ‘identity-based’ regulation could be particularly vital, providing a more resilient framework that anchors the behavior to the individual’s core values and standards [43].

Unlike extrinsic motivation, which relies on external rewards or punishments, intrinsic motivation sustains long-term engagement. According to our findings, sufficiently active patients demonstrated greater autonomous behavior in BREQ-3 scale scores. Intrinsic motivation emerged as a statistically significant associate of MVPA, explaining approximately 20% of its variance. While this proportion might be perceived as relatively low, it is consistent with the multifaceted nature of PA behavior in individuals with complex mental health conditions such as BD. Although this leaves a significant portion of the variance unexplained, a medium-to-large effect size was observed, highlighting the clinical relevance of internal drive for this population. This finding likely reflects the complex, multi-determined nature of exercise in BD, which is influenced by non-motivational factors such as medication side effects, mood fluctuations, and environmental barriers (2,3). Thus, while intrinsic motivation is a key associate, it should be viewed as one component of a broader biopsychosocial framework governing PA in patients with BD.

These results align with SDT principles, which emphasize that behaviors driven by interest, enjoyment, and inherent satisfaction are more likely to be sustained over time [7]. Recent evidence in psychiatric populations supports this, indicating that autonomous motivation – rather than controlled motivation (pressure or guilt) – is the primary driver of long-term PA adherence [9,30,41]. For instance, studies have shown that fostering a sense of competence through tailored exercise programs can mitigate the ‘amotivation’ often seen during depressive episodes [30]. Thus, our results reinforce that interventions focusing on the “why” of exercise, rather than just the “how much”, are essential for this population. This is particularly relevant in clinical populations, as intrinsic motivation can buffer against declines in motivation caused by mood fluctuations and symptoms, as often observed in BD individuals [44]. Furthermore, individuals with greater knowledge of their disorder and the health benefits of PA may be more inclined to integrate such behaviors into their lives for the long term [9].

The practical implications of our findings suggest that clinical interventions for BD should move beyond simply recommending exercise and instead focus on strategies that enhance autonomous motivation. Clinicians can foster this by supporting patients’ basic psychological needs – autonomy, competence, and relatedness – perhaps through motivational interviewing or by allowing patients to choose activities they find inherently enjoyable rather than prescribed routines. Regarding future research, longitudinal studies are needed to establish the temporal stability of these motivational regulations across different mood episodes. Additionally, investigating the role of social support and the impact of specific medication classes on the relationship between motivation and physical activity would provide a more nuanced understanding of how to sustain long-term exercise adherence in this population.

While our study offers novel insights into the motivational dynamics of Brazilian outpatients with BD, some limitations must be acknowledged. Generalizability across the broader population is curtailed by the modest sample size. Furthermore, this study may be subject to selection bias (due to convenience sampling), issues regarding the representativeness of the sample (predominance of female participants), and the implications of the cross-sectional design for causal inference. Moreover, regarding mood states and those sufficiently active patients, the majority in this study were manic, which could influence the motivational scores. Further, the majority of the insufficiently active patients were euthymic and depressive, and these mood states could influence motivational answers and scores. However, being the first Brazilian study to specifically examine motivational regulation in this clinical group, this study represents an important step toward culturally tailored and theory-based interventions. Another strength is the use of objective accelerometer data, enabling accurate quantification of PA – an advancement over self-reported measures.

## 5. Conclusion

In conclusion, our findings suggest that more autonomous forms of motivation, particularly integrated and intrinsic regulation, were associated with higher levels of PA among individuals with BD, whereas less active participants tended to present higher depressive symptomatology and lower motivational quality. These findings support the relevance of motivational processes proposed by SDT for understanding PA behaviors in this population and reinforce the importance of considering psychological and clinical factors when examining engagement in PA among individuals with BD. In addition, the predominance of externally regulated or less self-determined motivational profiles among insufficiently active participants may indicate important challenges for long-term adherence to PA. Future research should further investigate how SDT-related constructs interact with clinical characteristics, mood symptoms, and behavioral outcomes in individuals with BD, particularly in longitudinal and intervention-based contexts.

## Supporting information

S1 Fig *Behavioral Regulation Exercise Questionnaire – 3 (BREQ-3)* constructs: Amotivation (TIF)

S2 Fig *Behavioral Regulation Exercise Questionnaire – 3 (BREQ-3)* constructs: External regulation (TIF)

S3 Fig *Behavioral Regulation Exercise Questionnaire – 3 (BREQ-3)* constructs: Introjected regulation (TIF)

S4 Fig *Behavioral Regulation Exercise Questionnaire – 3 (BREQ-3)* constructs: Identified regulation (TIF)

S5 Fig *Behavioral Regulation Exercise Questionnaire – 3 (BREQ-3)* constructs: Integrated regulation (TIF)

S6 Fig *Behavioral Regulation Exercise Questionnaire – 3 (BREQ-3)* constructs: Intrinsic regulation (TIF)

S1 File PLOS_Human_Participants_Research_Checklist_2025 (PDF)

## References

[1] Vancampfort D, Rosenbaum S, Probst M, Connaughton J, du Plessis C, Yamamoto T, et al. Top 10 research questions to promote physical activity in bipolar disorders: A consensus statement from the International Organization of Physical Therapists in Mental Health. J Affect Disord. 2016;195:82–7. doi: 10.1016/j.jad.2016.01.046 26874245

[2] Voelker R. What is bipolar disorder? Bipolar disorder is characterized by mood swings ranging from depressive lows to manic highs. JAMA. 2024;331(10):894. doi: 10.1001/jama.2023.24844 38363576

[3] McIntyre RS, Berk M, Brietzke E, Goldstein BI, López-Jaramillo C, Kessing LV, et al. Bipolar disorders. Lancet. 2020;396(10265):1841–56. doi: 10.1016/S0140-6736(20)31544-0 33278937

[4] Castro Monteiro F, de Oliveira Silva F, Josiane Waclawovsky A, Ferreira JVA, Jesus-Moraleida FR, Schuch FB, et al. Physical activity and sedentary behavior levels among individuals with mental illness: A cross-sectional study from 23 countries. PLoS One. 2024;19(4):e0301583. doi: 10.1371/journal.pone.0301583 38669303 PMC11051624

[5] Vancampfort D, Firth J, Schuch FB, Rosenbaum S, Mugisha J, Hallgren M, et al. Sedentary behavior and physical activity levels in people with schizophrenia, bipolar disorder and major depressive disorder: A global systematic review and meta-analysis. World Psychiatry. 2017;16(3):308–15. doi: 10.1002/wps.20458 28941119 PMC5608847

[6] Firth J, Rosenbaum S, Stubbs B, Gorczynski P, Yung AR, Vancampfort D. Motivating factors and barriers towards exercise in severe mental illness: A systematic review and meta-analysis. Psychol Med. 2016;46(14):2869–81. doi: 10.1017/S0033291716001732 27502153 PMC5080671

[7] Deci EL, Ryan RM. The “what” and “why” of goal pursuits: Human needs and the self-determination of behavior. Psychol Inq. 2000;11(4):227–68. doi: 10.1207/S15327965PLI1104_01

[8] Vancampfort D, Madou T, Moens H, De Backer T, Vanhalst P, Helon C, et al. Could autonomous motivation hold the key to successfully implementing lifestyle changes in affective disorders? A multicentre cross sectional study. Psychiatry Res. 2015;228(1):100–6. doi: 10.1016/j.psychres.2015.04.021 25956760

[9] Vancampfort D, Moens H, Madou T, De Backer T, Vallons V, Bruyninx P, et al. Autonomous motivation is associated with the maintenance stage of behaviour change in people with affective disorders. Psychiatry Res. 2016;240:267–71. doi: 10.1016/j.psychres.2016.04.005 27131627

[10] Chapman J, Korman N, Malacova E, Robertson C, Arnautovska U, Siskind D, et al. Which behavioral regulations predict physical activity and sedentary behavior in people with mental illness?. Psychol Med. 2024;54(15):4129–39. doi: 10.1017/S0033291724001879 39569877 PMC11650172

[11] Seymour J, Pratt G, Patterson S, Korman N, Rebar A, Tillston S, et al. Changes in self-determined motivation for exercise in people with mental illness participating in a community-based exercise service in Australia. Health Soc Care Community. 2022;30(5):e1611–24. doi: 10.1111/hsc.13588 34614232

[12] Solmi M, Basadonne I, Bodini L, Rosenbaum S, Schuch FB, Smith L, et al. Exercise as a transdiagnostic intervention for improving mental health: An umbrella review. J Psychiatr Res. 2025;184:91–101. doi: 10.1016/j.jpsychires.2025.02.024 40043589

[13] Hird EJ, Slanina-Davies A, Lewis G, Hamer M, Roiser JP. From movement to motivation: A proposed framework to understand the antidepressant effect of exercise. Transl Psychiatry. 2024;14(1):273. doi: 10.1038/s41398-024-02922-y 38961071 PMC11222551

[14] Guedes D, Sofiati S. Tradução e validação psicométrica do Behavioral Regulation in Exercise Questionnaire para uso em adultos brasileiros. Rev Bras Ativ Fís Saúde. 2015;20(4):397. doi: 10.12820/rbafs.v.20n4p397

[15] Janney CA, Fagiolini A, Swartz HA, Jakicic JM, Holleman RG, Richardson CR. Are adults with bipolar disorder active? Objectively measured physical activity and sedentary behavior using accelerometry. J Affect Disord. 2014;152–154:498–504. doi: 10.1016/j.jad.2013.09.009 24095103 PMC3905833

[16] Amorim P. Mini International Neuropsychiatric Interview (MINI): Validação de entrevista breve para diagnóstico de transtornos mentais. Revista Brasileira de Psiquiatria. 2000;22(3):106–15. doi: 10.1590/s1516-44462000000300003

[17] Cronholm B, Schalling D, Asberg M. Development of a rating scale for depressive illness. Mod Probl Pharmacopsychiatry. 1974;7(0):139–50. doi: 10.1159/000395073 4412781

[18] Young RC, Biggs JT, Ziegler VE, Meyer DA. A Rating Scale for Mania. British Journal of Psychiatry. 1978;133:429–35. 728692 10.1192/bjp.133.5.429

[19] Schilling R, Cody R, Kreppke J-N, Faude O, Beck J, Brand S, et al. Correspondence between the Simple Physical Activity Questionnaire (SIMPAQ) and accelerometer-based physical activity in inpatients treated for major depressive disorders in comparison to non-depressed controls. Front Sports Act Living. 2024;6:1447821. doi: 10.3389/fspor.2024.1447821 39308892 PMC11412836

[20] Rosenbaum S, Morell R, Abdel-Baki A, Ahmadpanah M, Anilkumar TV, Baie L, et al. Assessing physical activity in people with mental illness: 23-country reliability and validity of the simple physical activity questionnaire (SIMPAQ). BMC Psychiatry. 2020;20(1):108. doi: 10.1186/s12888-020-2473-0 32143714 PMC7060599

[21] Troiano RP, Berrigan D, Dodd KW, Mâsse LC, Tilert T, McDowell M. Physical activity in the United States measured by accelerometer. Med Sci Sports Exerc. 2008;40(1):181–8. doi: 10.1249/mss.0b013e31815a51b3 18091006

[22] Ruiz JR, Ortega FB, Martínez-Gómez D, Labayen I, Moreno LA, De Bourdeaudhuij I, et al. Objectively measured physical activity and sedentary time in European adolescents. Am J Epidemiol. 2011;174(2):173–84. doi: 10.1093/aje/kwr068 21467152

[23] Herrmann SD, Barreira TV, Kang M, Ainsworth BE. How many hours are enough? Accelerometer wear time may provide bias in daily activity estimates. J Phys Act Health. 2013;10(5):742–9. doi: 10.1123/jpah.10.5.742 23036822

[24] Cleland V, Crawford D, Baur LA, Hume C, Timperio A, Salmon J. A prospective examination of children’s time spent outdoors, objectively measured physical activity and overweight. Int J Obes (Lond). 2008;32(11):1685–93. doi: 10.1038/ijo.2008.171 18852701

[25] Freedson PS. Calibration of the computer science and applications, inc. accelerometer. Med Sci Sports Exerc. 1998;30(5):777–81. 9588623 10.1097/00005768-199805000-00021

[26] Sasaki JE, John D, Freedson PS. Validation and comparison of ActiGraph activity monitors. J Sci Med Sport. 2011;14(5):411–6. doi: 10.1016/j.jsams.2011.04.003 21616714

[27] Sasaki J, Coutinho A, Santos C, Bertuol C, Minatto G, Berria J. Orientações para utilização de acelerômetros no Brasil. Revista Brasileira de Atividade Física & Saúde. 2017;22(2):110–26. doi: 10.12820/rbafs.v.22n2p110-126

[28] Rowlands AV. Accelerometer assessment of physical activity in children: An update. Pediatr Exerc Sci. 2007;19(3):252–66. doi: 10.1123/pes.19.3.252 18019585

[29] Vancampfort D, De Hert M, Vansteenkiste M, De Herdt A, Scheewe TW, Soundy A, et al. The importance of self-determined motivation towards physical activity in patients with schizophrenia. Psychiatry Res. 2013;210(3):812–8. doi: 10.1016/j.psychres.2013.10.004 24182688

[30] Vancampfort D, Stubbs B, Venigalla SK, Probst M. Adopting and maintaining physical activity behaviours in people with severe mental illness: The importance of autonomous motivation. Prev Med. 2015;81:216–20. doi: 10.1016/j.ypmed.2015.09.006 26386141

[31] Siopa C, Calaça M, Pestana PC, Novais F. Targeting neurotransmitter systems in bipolar disorder: A comprehensive Review of novel pharmacological approaches. Curr Treat Options Psych. 2024;11(3):188–202. doi: 10.1007/s40501-024-00327-8

[32] Walsh RFL, Smith LT, Klugman J, Titone MK, Ng TH, Goel N, et al. An examination of bidirectional associations between physical activity and mood symptoms among individuals diagnosed and at risk for bipolar spectrum disorders. Running Title: Physical Activity and Mood in Bipolar Disorder. 2023. 10.1016/j.brat.2023.104255 PMC9909602 36682182

[33] Tondo L, Miola A, Pinna M, Contu M, Baldessarini RJ. Differences between bipolar disorder types 1 and 2 support the DSM two-syndrome concept. Int J Bipolar Disord. 2022;10(1):21. doi: 10.1186/s40345-022-00268-2 35918560 PMC9346033

[34] Brancati GE, Nunes A, Scott K, O’Donovan C, Cervantes P, Grof P, et al. Differential characteristics of bipolar I and II disorders: A retrospective, cross-sectional evaluation of clinical features, illness course, and response to treatment. Int J Bipolar Disord. 2023;11(1):25. doi: 10.1186/s40345-023-00304-9 37452256 PMC10349025

[35] Faurholt-Jepsen M, Frost M, Busk J, Christensen EM, Bardram JE, Vinberg M. Differences in mood instability in patients with bipolar disorder type I and II: A smartphone-based study. Int J Bipolar Disord. 2019;7(1). doi: 10.1186/s40345-019-0141-4 PMC6355891 30706154

[36] Melo MCA, Garcia RF, de Araújo CFC, Rangel DM, de Bruin PFC, de Bruin VMS. Physical activity as prognostic factor for bipolar disorder: An 18-month prospective study. J Affect Disord. 2019;251:100–6. doi: 10.1016/j.jad.2019.03.061 30921592

[37] Monteiro FC, Schuch FB, Deslandes AC, Vancampfort D, Mosqueiro BP, Messinger MF, et al. Perceived barriers, benefits and correlates of physical activity in outpatients with Major Depressive Disorder: A study from Brazil. Psychiatry Res. 2020;284:112751. doi: 10.1016/j.psychres.2020.112751 31918115

[38] Firth J, Solmi M, Wootton RE, Vancampfort D, Schuch FB, Hoare E, et al. A meta-review of “lifestyle psychiatry”: the role of exercise, smoking, diet and sleep in the prevention and treatment of mental disorders. World Psychiatry. 2020;19(3):360–80. doi: 10.1002/wps.20773 32931092 PMC7491615

[39] McCartan CJ, Yap J, Best P, Breedvelt J, Breslin G, Firth J, et al. Factors that influence participation in physical activity for people with bipolar disorder: A synthesis of qualitative evidence. Cochrane Database Syst Rev. 2024;6(6):CD013557. doi: 10.1002/14651858.CD013557.pub2 38837220 PMC11152184

[40] van Rijen D, Ten Hoor GA. A qualitative analysis of facilitators and barriers to physical activity among patients with moderate mental disorders. Z Gesundh Wiss. 2022;:1–16. doi: 10.1007/s10389-022-01720-4 35668718 PMC9157478

[41] Teixeira PJ, Carraça EV, Markland D, Silva MN, Ryan RM. Exercise, physical activity, and self-determination theory: A systematic review. Int J Behav Nutr Phys Act. 2012;9:78. doi: 10.1186/1479-5868-9-78 22726453 PMC3441783

[42] Rhodes RE, Rebar AL, Strachan S. Physical activity identity as an axis of dual process motivation and self-regulation processes: Current evidence and future research directions. Psychol Sport Exerc. 2025;80:102923. doi: 10.1016/j.psychsport.2025.102923 40541819

[43] Wierts CM, Kroc E, Rhodes RE. The role of intention, behavioral regulation, and physical activity behavior in the prediction of physical activity identity across time. Behav Sci (Basel). 2024;14(10):886. doi: 10.3390/bs14100886 39457757 PMC11505567

[44] Bauer IE, Kiropoulos LA, Crist NP, Hamilton JE, Soares JC, Meyer TD. A qualitative study investigating bipolar patients’ expectations of a lifestyle intervention: A self-management program. Arch Psychiatr Nurs. 2018;32(4):555–60. doi: 10.1016/j.apnu.2018.03.002 30029747

